# Supplementary material for: ketu mutant mice uncover an essential meiotic function for the ancient RNA helicase YTHDC2
Source: eLife. 2018 Jan 23;7:e30919. doi: 10.7554/eLife.30919 (PMC5832417; doi:10.7554/eLife.30919)
Supplement: Transparent reporting form [file elife-30919-transrepform.docx]

***eLife’s* transparent reporting form**

We encourage authors to provide detailed information *within their submission* to facilitate the interpretation and replication of experiments. Authors can upload supporting documentation to indicate the use of appropriate reporting guidelines for health-related research (see [EQUATOR Network](http://www.equator-network.org/%20)), life science research (see the [BioSharing Information Resource](https://biosharing.org/)), or the [ARRIVE guidelines](http://www.plosbiology.org/article/info:doi/10.1371/journal.pbio.1000412) for reporting work involving animal research. Where applicable, authors should refer to any relevant reporting standards documents in this form.

If you have any questions, please consult our Journal Policies and/or contact us: [editorial@elifesciences.org](mailto:editorial@elifesciences.org).

**Sample-size estimation**

- You should state whether an appropriate sample size was computed when the study was being designed
- You should state the statistical method of sample size computation and any required assumptions
- If no explicit power analysis was used, you should describe how you decided what sample (replicate) size (number) to use

Please outline where this information can be found within the submission (e.g., sections or figure legends), or explain why this information doesn’t apply to your submission:

For screening, ≥ 24 G3 mice were assessed and considered sufficient to yield ≥ 1 potential mutants with matching phenotypes based on Mendelian segregation. For mutation mapping and variant discovery of the *ketu* mutant line, 5 G3 mice were analyzed and considered sufficient to yield a single region of shared B6 homozygosity and high confidence variant calling, respectively.

Results from the initial characterization of the *Ythdc2^ketu^* mutants generated from our screen (hypogonadism, infertility, presence of abnormal metaphases, lack of developing follicles in females) were confirmed in *Ythdc2^em1^* mice generated using CRISPR/Cas9.

For most cytological and histological analyses, ≥ 2 animals of similar ages were evaluated and deemed sufficient to assess reproducibility of phenotypes.

Transcriptome analyses by RNA sequencing were done as a time-course experiment with ≥ 2 animals per age, which was deemed sufficient to ensure correct interpretation of transcriptome changes.

Biochemistry assays were performed in triplicates and most assays were validated by repeating experiments with independent protein preps.

All relevant information is stated within the text, Figures or Figure legends.

**Replicates**

- You should report how often each experiment was performed
- You should include a definition of biological versus technical replication
- The data obtained should be provided and sufficient information should be provided to indicate the number of independent biological and/or technical replicates
- If you encountered any outliers, you should describe how these were handled
- Criteria for exclusion/inclusion of data should be clearly stated
- High-throughput sequence data should be uploaded before submission, with a private link for reviewers provided (these are available from both GEO and ArrayExpress)

Please outline where this information can be found within the submission (e.g., sections or figure legends), or explain why this information doesn’t apply to your submission:

Biological replicates refer to independent mice/protein preparations and technical replicates refer to independent samples from the same mouse/protein preparation.

Screening data in **Figure 1C**,**D** include ≥ 3 biological replicates and one technical replicate. Hypogonadism data in **Figure 3A** include ≥ 3 biological replicates.

Cytological data in **Figure 1B**,**E** and **Figure 4F**,**G** are representative of ≥ 2 biological replicates of similar ages and ≥ 2 technical replicates.

All histological data are representative of ≥ 2 technical replicates. Most histological data are representative of ≥ 2 biological replicates of similar ages. Histological data in **Figure 4−Figure Supplement 1B** represent one biological replicate. Histological data of adults in **Figure 7B** and **Figure 7−Figure Supplement 1A** represent one biological replicate and independent 14 *dpp*-old animals gave similar results.

All biochemistry assays are representative of 3 technical replicates. Biochemistry assays with wild-type protein are representative of two biological replicates of differing constructs and those with mutant are representative of one biological replicate.

Exome sequencing was done with 5 biological replicates and RNA sequencing was done with ≥ 2 biological replicates per age. ENCODE data in **Figure 2D** are from 2 biological replicates.

For assessing YTHDC2 localization in wild-type mice (**Figure 7C** and **Figure 7−Figure Supplement 1B**), immunofluorescence patterns that were not reproduced by both antibodies that we tested were considered unreliable and excluded.

All relevant information is stated within the text, Figures or Figure legends.

**Statistical reporting**

- Statistical analysis methods should be described and justified
- Raw data should be presented in figures whenever informative to do so (typically when N per group is less than 10)
- For each experiment, you should identify the statistical tests used, exact values of N, definitions of center, methods of multiple test correction, and dispersion and precision measures (e.g., mean, median, SD, SEM, confidence intervals; and, for the major substantive results, a measure of effect size (e.g., Pearson's r, Cohen's d)
- Report exact p-values wherever possible alongside the summary statistics and 95% confidence intervals. These should be reported for all key questions and not only when the p-value is less than 0.05.

Please outline where this information can be found within the submission (e.g., sections or figure legends), or explain why this information doesn’t apply to your submission:

Statistical tests conducted and p-values obtained are stated within the text. Exact values of n are stated within Figures or Figure legends. Raw data are provided in Source Data files.

(For large datasets, or papers with a very large number of statistical tests, you may upload a single table file with tests, Ns, etc., with reference to sections in the manuscript.)

**Group allocation**

- Indicate how samples were allocated into experimental groups (in the case of clinical studies, please specify allocation to treatment method); if randomization was used, please also state if restricted randomization was applied
- Indicate if masking was used during group allocation, data collection and/or data analysis

Please outline where this information can be found within the submission (e.g., sections or figure legends), or explain why this information doesn’t apply to your submission:

Data in **Figure 1C**,**D** were assessed and scored blind. For all other experiments, individual mice were analyzed and samples were grouped by genotype for statistics.

All relevant information is stated within the text, Figures or Figure legends.

**Additional data files (“source data”)**

- We encourage you to upload relevant additional data files, such as numerical data that are represented as a graph in a figure, or as a summary table
- Where provided, these should be in the most useful format, and they can be uploaded as “Source data” files linked to a main figure or table
- Include model definition files including the full list of parameters used
- Include code used for data analysis (e.g., R, MatLab)
- Avoid stating that data files are “available upon request”

Please indicate the figures or tables for which source data files have been provided:

RNA seq data are available from GEO.

The following supplementary files have been provided: **Supplementary File** **1** (Expression fold changes of differentially expressed genes in **Figure 6B**), **Supplementary File** **2** (Protein accession numbers for **Figure 8A**), **Supplementary File 3** (Protein accession numbers for **Figures 8B–D**, **10** and **11**) and **Supplementary File 4** (Genotyping primers and oligos used to make helicase assay substrates).

Source data files have been provided for **Figure 1D**, **Figure 2D**, **Figure 3A**, **Figure 3−Figure Supplement 1A**, **Figure 4D**, **Figure 4E**, **Figure 5B**, **Figure 5C**, **Figure 5E**, **Figure 5F**, **Figure 5−Figure Supplement 1**, **Figure 6C**, **Figure 6D**, and **Figure 6E**.
